# The Role of Paramedics in Diagnosing Sandifer’s Syndrome

**DOI:** 10.3390/healthcare13080883

**Published:** 2025-04-11

**Authors:** Michał Wójcik, Damian Krysiak, Piotr Babik, Łukasz Suchanek, Michał Ćwiertnia, Joanna Trojak-Piętka, Marek Kawecki, Wioletta Pollok-Waksmańska, Monika Mikulska, Tomasz Ilczak

**Affiliations:** 1 Department of Emergency Medicine, Faculty of Health Sciences, University of Bielsko-Biala, 43-309 Bielsko-Biała, Poland; dkrysiak@ubb.edu.pl (D.K.); pbabik@ubb.edu.pl (P.B.); lsuchanek@ubb.edu.pl (Ł.S.); mcwiertnia@ubb.edu.pl (M.Ć.); jpietka@ubb.edu.pl (J.T.-P.); mkawecki@ubb.edu.pl (M.K.); mmikulska@ubb.edu.pl (M.M.); tilczak@ubb.edu.pl (T.I.); 2 European Pre-Hospital Research Network, Nottingham NG11 8NS, UK; 3 Department of Public Health, Faculty of Health Sciences, University of Bielsko-Biala, 43-309 Bielsko-Biała, Poland; wwaksmanska@ubb.edu.pl

**Keywords:** Sandifer’s syndrome, paediatric, seizures, gastroesophageal reflux, emergency medical service, vagus nerve

## Abstract

**Background:** Sandifer’s syndrome is an uncharacteristic symptom of gastroesophageal reflux disease (GERD). It is often misdiagnosed as epilepsy. Paramedics can play a crucial role in recognising the differences between Sandifer’s syndrome and epilepsy. Therefore, education is important to reduce the likelihood of misdiagnosis and the mistreatment of patients. This purpose of this study is to provides information and guidelines for collecting patients’ medical history and identifying the most common symptoms, which support pre-hospital suspicion of Sandifer’s syndrome. **Methods:** The study consisted of a clinical case study, concerning the management of the emergency team, in a 7-week-old child with symptoms indicative of an epileptic seizure. **Results:** The clinical case analysis showed that a thorough examination of the patient helped to rule out epilepsy in the child and observed the characteristic symptoms of Sandifer syndrome. While assisting the child, a rare symptom of apnoea was also observed. **Conclusions**: The role of the paramedics in diagnosing Sandifer’s syndrome can be crucial. Their experience and knowledge of emergency situations, as well as correctly conducted tests during and immediately after ailment symptoms, can provide medical teams with key information that can help in making a correct diagnosis. The presented framework can be helpful. In the majority of cases, a correct diagnosis leads to the complete cessation of symptoms and lowers the risk of side effects from unnecessarily applied anti-epilepsy medication.

## 1. Introduction

According to statistics from the Polish Central Statistical Office, emergency response teams (ERTs) intervened almost 3.1 million times in 2022 [1]. Interventions involving children and adolescents below 18 years of age constituted only 6.7% of this figure, which corresponds to European estimates, accounting for 6–10% of all emergency department patients [2]. Research conducted in simulated conditions on a group of paramedics working in Switzerland confirmed that interventions which involve providing assistance to children are highly stressful for medical personnel. This may be related to time pressure, limited experience with paediatric patients, and, in particular, the need to calculate and properly administer specific drug doses [3]. It is estimated that chronic diseases in paediatric patients affect 30% of children around the world [4]. According to various sources, gastroesophageal reflux affects, depending on age, around 10% of children, and among infants, this percentage rises even to 80 to 90%. Sandifer’s syndrome, which is a non-specific set of symptoms, affects only 1% of these children [5]. Due to the atypical symptoms suggesting a problem of neurological and not gastrological origin, it may be the reason for requesting an ERT intervention and can be connected to unnecessary pharmacotherapy, erroneous procedures, and extended diagnosis. A thorough examination and the collection of a detailed medical history can help members of an emergency medical team to make the correct diagnosis and implement the right management [5].

The aim of this article is to present a rare disease entity occurring among children, which is Sandifer’s syndrome. The article presents the difficulties that emergency medical teams may encounter when providing assistance to a patient with this disease.

## 2. Gastroesophageal Reflux and Gastroesophageal Reflux Disease

Gastroesophageal reflux (GER) is a very common phenomenon, affecting, depending on the source, from about half to even two-thirds of all infants [6,7]. The backflow of stomach contents into the oesophagus is a physiological process that occurs several times a day in healthy infants, children, and adults [7]. The causes vary with age. In older children and adults, dysfunction in the lower oesophageal sphincter is the primary factor, while in infancy, the pressure difference between the stomach and oesophagus also plays a significant role [8]. In 2009, the North American Society for Pediatric Gastroenterology, Hepatology, and Nutrition (NASPGHAN) and the European Society for Paediatric Gastroenterology, Hepatology, and Nutrition (ESPGHAN) issued a joint statement distinguishing gastroesophageal reflux disease (GERD) from GER (despite these terms often being used interchangeably). GERD is defined as the occurrence of troublesome symptoms and complications resulting from GER [7,9]. In children, particularly those who have not yet developed verbal communication skills, it is often impossible to determine whether “troublesome symptoms” are present. Therefore, different symptoms of GERD are assigned depending on the patient’s age. The most common symptoms in infants include recurrent vomiting, feeding aversion, frequent choking, as well as irritability and frequent crying. In adults and older children, the primary symptoms include recurrent vomiting, abdominal or chest pain with a burning sensation, primarily heartburn, and swallowing disorders (dysphagia) [7,9]. Since 2009, the definition has not been updated. Based on this definition, guidelines were developed, initially by the National Institute for Health and Care Excellence (NICE) in 2015, and later updated in 2018 with joint recommendations by the NASPGHAN and ESPGHAN [9,10].

## 3. What Is Sandifer’s Syndrome and What Are the Symptoms?

The illness currently known as Sandifer’s syndrome was first described in the 1960s by the Austrian neurologist Marcel Kinsbourn. It takes its name from a British doctor, Paul Sandifer, who was the first to notice a correlation between the occurrence of torticollis in children after a meal [11,12]. According to the definition, it is a non-specific, extraoesophageal manifestation of gastroesophageal reflux disease (GERD), accompanied by neurological symptoms similar to epileptic seizures [12]. In the literature, it is most commonly described as dystonia, the occurrence of involuntary movements located around the head, neck, and torso, as well as shaking, problems with breathing, eye rotation and a decrease in muscle tone [11,13]. In contrast to epileptic seizures, during seizures in Sandifer’s syndrome, there is no rhythmic or clonic component, and the symptoms do not affect the limbs [5,11,12]. In addition, symptomatic episodes do not occur during sleep, and patients are conscious and partially react to stimuli [5]. The next important piece of information that makes it possible to distinguish the syndrome is the time when seizures occur: usually a short time after feeding [14]. In addition to the symptoms ascribed to Sandifer’s syndrome, depending on age, children will have various gastroesophageal reflux disease (GERD) symptoms. In infants, these include crying and irritation, unwillingness to feed, and further consequences involving problems with gaining weight and recurrent respiratory tract infections, including lung infections [15]. In older children, the symptoms are identical to those that occur in adults, that is, burping, heartburn, vomiting, a chronic cough, chronic stridor and wheezing, recurrent lung infections, and apnoea [5,15].

## 4. Aetiology

Despite being recognised for many years, the exact cause of these symptoms remains unknown. Some authors suggest that dystonia and the body position are adopted by children unconsciously and that it is a learned behaviour that aims to ease discomfort at the moment of gastric reflux [5]. It has been proven that the adopted position accelerates the process of emptying the stomach [16]. However, this explains the occurrence of only some of the symptoms. The latest hypothesis is based on the position that it is a response of the vagus nerve, with its nerve endings being irritated by the released acidic content [16]. The vagus nerve is the longest cranial nerve and controls the most important functions of the human body. These include heart rate, blood pressure, breathing, and intestinal motility, as well as swallowing, the cough reflex, and the immunological response as a reaction to disease [17]. It is also responsible for the functioning of some muscles [17]. The vagus nerve’s fibres consist of both sensory neurons (around 80%) and motor and parasympathetic neurons. It is responsible for innervation of the throat, larynx, and heart, parts of the respiratory tract including the lungs, and parts of the digestive tract: the oesophagus, stomach, liver, pancreas, small intestine, and proximal colon [18,19]. There is ongoing research and debate as to whether this does not also include the spleen, kidneys, adrenal glands, and reproductive organs. It continues to be the subject of many discussions. The complex structure of the vagus nerve allows for the implementation of therapies in patients, acting on it to treat various medical conditions [19]. The most common example is the stimulation of this nerve during supraventricular tachycardia, which is used to slow the heart rate. Increasingly, targeted electrical impulses are being used to treat a variety of clinical disorders, such as heart failure, migraine, and inflammatory bowel disease [17]. Given the complexity of the vagus nerve and the nonspecific, often seemingly unrelated symptoms of Sandifer’s syndrome, it seems justified to search for the cause in its irritation.

## 5. Diagnostics and Treatment

In medical ERT practice, it is not possible to make a clear diagnosis due to limited diagnostic capabilities. However, due to the fact that an ERT is called for at the moment when symptoms appear, paramedics are able to notice the difference between symptoms of Sandifer’s syndrome and those of typical epileptic seizures, which is not possible in the case of planned visits to specialist clinics. An additional difficulty in making a correct diagnosis is that the child’s parents often do not have specialist medical knowledge, and they are also subjected to the additional factor of stress when the symptoms appear. Then, interviewing them is incomplete, chaotic and not very accurate. An additional difficulty is the impossibility of interviewing the child directly. This often directs diagnosis and treatment towards epileptic seizures [13]. This involves costly tests of the nervous system in which deviations from the norm are not found, extending the time taken to give the correct diagnosis and apply appropriate treatment, and above all, the use of antiepileptic medicines that do not have a neutral effect on the body may occur. In research conducted by Kotagala et al. [20] from the university of Cleveland in 1989–1995, among a group of 883 children on an epilepsy monitoring ward, 134 children were found not to have epilepsy. In the youngest age group (2–5 years), Sandifer’s syndrome was one of the three mostly frequently diagnosed diseases. Therefore, differential diagnosis performed in a hospital or in a specialist clinic is very important. In the initial diagnostic stage, medical interviews play a crucial role. Various questionnaires have been created to assess the frequency and intensity of symptoms, such as the Gastroesophageal Reflux Disease Health-Related Quality of Life (GERD-HRQL) Questionnaire or the Gastrointestinal Symptom Rating Scale (GSRS) [21]. These are effective methods for conducting subjective assessments but may be ineffective in the youngest patients. Nonetheless, it is believed that a well-conducted medical interview is sufficient to confirm GERD, and additional tests such as contrast radiography of the upper gastrointestinal tract, esophageal pH monitoring, gastroesophageal scintigraphy, or esophageal endoscopy with biopsy are not necessary. However, there may be additional concerning symptoms that require extended diagnostics. These include gastrointestinal bleeding, hepatosplenomegaly, persistent and severe vomiting, fever, abnormal head size, and bulging fontanelle [7]. Differentiating Sandifer’s syndrome from epileptic seizures is primarily based on EEG recordings and neuroimaging diagnostics of the central nervous system. Once a diagnosis has been made, the cure is based on treatment of GERD, after which the symptoms of Sandifer’s syndrome disappear. Treatment does not only consist of pharmacotherapy; initially, a modification to the diet is applied as well as education for the parents regarding feeding habits. An example of this is not giving the child excessively large meals and preventing the child from adopting a reclining position immediately after food [5]. Only when non-pharmacological methods do not bring results should pharmacological treatment be applied. The principal method is to use proton pump inhibitors and H2 blockers, after which, in most cases, the symptoms disappear [5,13]. Only in a few cases is surgical intervention or enteral feeding necessary [13]. To date, no recommendations and guidelines have been developed anywhere in the world for emergency response teams. In practice, when a patient has confirmed Sandifer’s syndrome, a crucial part of the procedure is the appropriate examination of the child to exclude other emergency conditions, including, above all, epileptic seizures, as well as ceasing the administration of benzodiazepine and the possible implementation of treatment for accompanying symptoms. If breathing problems occur, it may be necessary to assist with breathing. The patient should always be transported to hospital. This will enable broader diagnosis and possible modification to the treatment implemented so far [13].

## 6. Case Study Description

The parents of an unconscious seven-week-old boy, weighing approximately 5500 g, returned to the emergency medical service (EMS) station. The reason for their visit was their unsuccessful attempts to wake the boy, which lasted about 10 min. Due to the short distance from their home to the EMS station, the parents decided to seek help on their own. One member the EMS team immediately assessed the patient’s condition following the ABCDE protocol recommended by the European Resuscitation Council guidelines, while the other went to obtain the equipment needed to conduct paediatric advanced life support. The assessment of airway patency revealed no obstruction or risk of obstruction. In response to a painful stimulus, the boy displayed a facial grimace. He also briefly opened his eyes. His respiratory rate was approximately 30 breaths per minute, with no signs of respiratory distress, accessory muscle use, nasal flaring, or abnormal breath sounds. Auscultation revealed symmetrical physiological vesicular breath sounds over the lung fields. His heart rate was regular, with distinct heart sounds and a palpable brachial pulse at approximately 140 beats per minute. His blood pressure was within the normal range for his age. The examination showed that the child’s skin was pink, adequately moist, and of a normal temperature, with a capillary refill time of less than 2 s. The tympanic membrane temperature measured 36.8 °C. During the patient examinations, a medical history was obtained from the child’s parents. From the conversation, it was found that the child had been discharged from the neurology department of a children’s hospital a few days earlier after previously staying in intensive care due to suspected epilepsy. The mother had the hospital discharge summary and handed it to the paramedics. During hospitalisation, imaging studies (MRI, ultrasound), laboratory tests, and functional tests (EEG) were performed, all of which showed no pathological disorder, effectively ruling out epilepsy The attending neurologist, based on the conducted diagnostics, referred the child to a neurology and gastroenterology clinic upon discharge. The referral to the gastroenterology clinic was mainly due to the doctor’s suspicion of Sandifer’s syndrome. The parents, following recommendations, contacted a gastroenterology clinic and were awaiting their scheduled appointment. Neither the child nor the mother were on any long-term medications, and the child had been breastfed a maximum of two hours before falling asleep. During the medical history assessment, the paramedics observed a sudden change in the child’s body position, involving the head, neck, and torso. The child’s body was arched in a posture resembling an inverted “U”. The paramedics noted no increased muscle tone in the limbs. Moments later, they observed that the child’s chest stopped rising. The child’s breathing was reassessed and found to have ceased. One of the EMS team members, acting as the team leader, instructed the second paramedic to prepare a self-inflating bag. While preparing the equipment, the child was listened to again and no vesicular murmur was found, accompanied by a continued regular heartbeat and no drop in oxygen saturation. The child’s skin remained pink, warm, and properly hydrated. A painful stimulus was applied to stimulate breathing, but without effect. After approximately 30 s, spontaneous respiration resumed at a rate of 30 to 35 breaths per minute, eliminating the need for assisted ventilation. The unnatural body posture disappeared along with the apnea, and the child began spontaneously opening his eyes. A neurological examination showed no abnormalities: muscle strength was symmetrically preserved, the child responded to pain stimuli with crying, no cranial nerve dysfunction was observed, and no signs of meningeal irritation or fever were present. From that moment on, the child’s condition remained unchanged. Based on the observed symptoms, collected medical history, and previous hospital tests, the EMS team confirmed the physicians’ suspicions, identifying characteristic features of Sandifer’s syndrome. The child was transported to the nearest paediatric hospital. During transport, the paramedic informed the hospital staff about the child’s condition and estimated time of arrival. Further observation and diagnostics conducted in the paediatric ward and gastroenterology clinic confirmed later the diagnosis of Sandifer’s syndrome, validating the EMS team’s initial suspicions.

## 7. Conclusions

**Figure 1:**
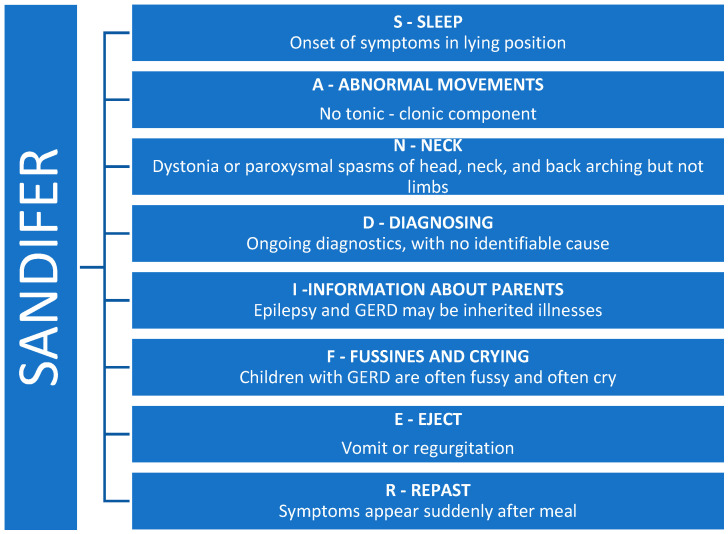
Scheme containing key information for considering diagnosis of Sandifer’s syndrome.

The role of paramedics in diagnosing Sandifer’s syndrome can be crucial. Their experience and knowledge of emergency situations, as well as correctly conducted tests during and immediately after ailment symptoms, can provide the team of doctors with key information that can help in making a correct diagnosis. To maximise the chances of making an accurate diagnosis, an acronym was created to identify the most common shared characteristics (Figure 1).

This pattern for conducting a medical interview is intended to serve as a guideline for conversations with the patient’s parents and should not be considered a substitute for clinical assessment or applied to all patients. The prehospital diagnosis of Sandifer’s syndrome requires a detailed medical history, including information about the type of food and time since the last feeding, the position in which the patient was in when symptoms appeared, chronic illnesses of the parents, and, in the case of breastfeeding, any medications taken by the mother. Key characteristic elements to consider include frequent regurgitation, feeding aversion, and the occurrence of symptoms after meals or in a lying position. Additionally, it is crucial to differentiate Sandifer’s syndrome from a typical epileptic seizure: the patient responds to stimulation (especially painful stimulation), there is no tonic–clonic component, and symptoms mainly affect the head, neck, and chest, without involving the limbs. These findings strongly suggest Sandifer’s syndrome. However, confirmation of the diagnosis requires additional in-hospital examination. The clinical case described presented not only the characteristic features of Sandifer’s syndrome but also the rarely described apnea, which lasted for nearly half a minute. In the majority of cases, a correct diagnosis leads to the complete cessation of symptoms and lowers the risk of side effects from unnecessarily applied anti-epilepsy medication and medical procedures, which occurred in this case. Therefore, the education of medical personnel should include topics related to the management of patients with Sandifer’s syndrome.

## Data Availability

The datasets used and/or analysed during the current study are available from the corresponding author on reasonable request.
